# Genetic association analysis of miRNA SNPs implicates *MIR145* in breast cancer susceptibility

**DOI:** 10.1186/s12881-015-0248-0

**Published:** 2015-11-17

**Authors:** Diego Chacon-Cortes, Robert A. Smith, Larisa M. Haupt, Rodney A. Lea, Philippa H. Youl, Lyn R. Griffiths

**Affiliations:** 1 Genomics Research Centre, Institute of Health and Biomedical Innovation, Queensland University of Technology, 60 Musk Avenue, Kelvin Grove, Queensland 4059 Australia; 2 Griffith Health Institute, Griffith University, Griffit, Queensland Australia; 3 Cancer Council Queensland, Brisbane, Queensland Australia; 4 School of Public Health, Queensland University of Technology, Brisbane, Queensland Australia

**Keywords:** Association analysis, Breast cancer, microRNA, miR-SNPs, MIR145

## Abstract

**Background:**

MicroRNAs (miRNAs) are important small non-coding RNA molecules that regulate gene expression in cellular processes related to the pathogenesis of cancer. Genetic variation in miRNA genes could impact their synthesis and cellular effects and single nucleotide polymorphisms (SNPs) are one example of genetic variants studied in relation to breast cancer. Studies aimed at identifying miRNA SNPs (miR-SNPs) associated with breast malignancies could lead towards further understanding of the disease and to develop clinical applications for early diagnosis and treatment.

**Methods:**

We genotyped a panel of 24 miR-SNPs using multiplex PCR and chip-based matrix assisted laser desorption ionization time-of-flight (MALDI-TOF) mass spectrometry (MS) analysis in two Caucasian breast cancer case control populations (Primary population: 173 cases and 187 controls and secondary population: 679 cases and 301 controls). Association to breast cancer susceptibility was determined using chi-square (*X*^*2*^) and odds ratio (OR) analysis.

**Results:**

Statistical analysis showed six miR-SNPs to be non-polymorphic and twelve of our selected miR-SNPs to have no association with breast cancer risk. However, we were able to show association between rs353291 (located in MIR145) and the risk of developing breast cancer in two independent case control cohorts (*p* = 0.041 and *p* = 0.023).

**Conclusions:**

Our study is the first to report an association between a miR-SNP in MIR145 and breast cancer risk in individuals of Caucasian background. This finding requires further validation through genotyping of larger cohorts or in individuals of different ethnicities to determine the potential significance of this finding as well as studies aimed to determine functional significance.

## Background

MicroRNAs (miRNAs) are small non-coding single stranded RNA molecules (21 – 25 nucleotides) involved in negative regulation of gene expression [1, 2]. miRNAs are transcribed from long microRNA primary transcripts (pri-miRNAs) containing miRNA precursors (pre-miRNA, stem-loop molecules of 55 – 70 nucleotides). Pre-miRNAs are usually generated via the canonical pathway [3], where pri-miRNAs are cleaved by the complex formed by the nuclear ribonuclease (RNase) III DROSHA and the RNA-binding protein DCGR8 (DiGeorge syndrome critical region gene 8) [4]. Following transport to the cytoplasm by XPO5 (exportin5) and the nuclear protein ran-GTP, they are processed by DICER1 [5, 6]. The resultant duplex molecule contains both a single-stranded mature miRNA sequence and a complementary miRNA* strand which is released and degraded while the mature miRNA is loaded into the RNA-induced silencing complex (RISC) and merged into the Argonaute/EIFC2C (Ago) proteins [7, 8]. Each miRNA may bind to up to 200 gene targets and multiple binding sites for different miRNAs, making a highly complex web of interactions affecting a variety of biological pathways [9, 10].

miRNAs are very likely to play an important role in cancer biology due to their role in the regulation of important cellular processes including growth, differentiation and cell survival [11–16]. Several mechanisms result in changes in miRNA synthesis and expression in relation to cancer including: point mutations in miRNA and mRNA sequences, loss or mutation in the promoter regions for specific miRNA clusters, epigenetic changes and alterations in pathway related to dsRBD proteins [17–19]. Single nucleotide polymorphisms (SNPs) in miRNA genes (miR-SNPs) are one example of point mutation (albeit one occurring in the past) that could affect miRNA function in one of three possible ways: altering transcription of the primary miRNA transcript; processing of the pri-miRNA and pre-miRNA and; through their effects on modulation of miRNA-mRNA interactions [20–23]. As a result miR-SNPs have been associated with different types of cancer, including chronic lymphocytic leukaemia, gastric, lung and thyroid carcinoma [24–27].

Breast cancer is the second most commonly diagnosed cancer worldwide (1.67 million new cases, 25%) and the most common type of cancer for women in developed countries (793,684 new cases, 28%) [28]. Recent evidence suggests a role for miR-SNPs in breast cancer susceptibility including work by Hu et al. where the presence of mutant alleles of MIR196A2 rs11614913 and MIR499A rs3746444 significantly increased breast cancer risk in Chinese women [29]. However in genetic association analysis of Caucasian populations and functional studies in breast cancer cell lines performed by Hoffman et al. the presence of SNP in MIR196A2 rs11614913 was significantly associated with reduced risk of breast cancer, as well as less efficient processing of MIR196A2 and reduced capacity to regulate target genes, indicating additional factors may be at work in this SNP’s effect on breast cancer [30]. Additionally, Kontorovich et al. found 2 SNPs, rs6505162 and rs895819 located in MIR423 and MIR27A precursors respectively, to be significantly associated with decreased risk of breast cancer in BRCA2 mutation carriers from a Jewish population [31]. rs895819 was also significantly associated with reduced risk of developing breast cancer in families with a history of non-BRCA related breast cancer in a later study performed by Yang et al [32]. Finally rs2910164, a miR-SNP located in the 3p strand of MIR146A, was found to be associated with a younger age of diagnosis in familial breast cancer for BRCA1 mutation carriers [33].

In this study, we have investigated a panel of miR-SNPs present in miRNA genes previously identified to be involved in the pathophysiological mechanisms of breast cancer. Our initial selection included 24 variants located in 10 miRNA genes or in close proximity which were genotyped using multiplex PCR and matrix-assisted laser desorption/ionisation time-of-flight mass spectrometry (MALDI-TOF MS) analysis. Genotyping results of the 19 miR-SNPs successfully genotyped and included in this study identified six previously reported microRNA SNPs as non-polymorphic. From the remaining polymorphisms twelve miR-SNPs showed no significant differences between cases and controls and one variant in MIR145 was identified to be associated with breast cancer susceptibility in both of our Australian Caucasian breast cancer case-control populations.

## Methods

### Study populations

Two independent Australian Caucasian breast cancer case populations were available for our study: The Genomics Research Centre Breast Cancer (GRC-BC) population and part of the Griffith University-Cancer Council Queensland Breast Cancer Biobank (GU-CCQ BB). We conducted single nucleotide polymorphism genotyping in the GRC-BC population initially. This consisted of DNA samples from 173 breast cancer patients from South East Queensland and DNA samples from 187 healthy age and sex matched females with no personal and/or familial history of breast, ovarian or any other type of cancer collected at the Genomics Research Centre Clinic, Southport, with research approved by Griffith University’s Human Ethics Committee (Approval: MSC/07/08/HREC and PSY/01/11/HREC) and the Queensland University of Technology Human Research Ethics Committee (Approval: 1400000104). Breast cancer samples comprised prevalent breast cancer cases diagnosed previous to their inclusion in this study. All participants supplied informed written consent. Average age of test population was 57.52 years and 57 years for cases and controls respectively.

Further validation of genotyping results was performed on a subset of the GU-CCQ BB population. 679 DNA samples from breast cancer patients residing in Queensland with a diagnosis of invasive breast cancer confirmed histologically were used to validate genotyping of miR-SNPs. Patient samples had been collected by the Genomics Research Centre in collaboration with the Cancer Council of Queensland as part of a 5-year population-based longitudinal study since January 2010. Patients included in this study were between 33 and 80 years of age, with an average age of 60.16 and they were screened for personal and/or familial history of breast, ovarian or any other type of cancer. Control population for the GU-CCQ BB was established from 2 sources: The control group for this cohort was comprised of genotyping result data taken from 201 healthy females belonging to the phase 1 European population from the 1000Genomes project. Efforts were made to select a subgroup of individuals that were comparable to the case group in terms of age, ethnicity and sex [34].

### Genomic DNA sample preparation from whole human blood

Genomic DNA was extracted from whole blood samples using a modified salting out method described previously [35, 36]. DNA samples were evaluated by spectrophotometry using the Thermo Scientific NanoDrop™ 8000 UV-Vis Spectrophotometer (Thermo Fisher Scientific Inc., Wilmington, DE. USA) to determine DNA yield and 260/280 ratios [37–39]. Samples with a reading below 1.7 for their 260/280 ratio were purified using an ethanol precipitation protocol to guarantee DNA sample purity [40].

### miRNA SNP selection

**Fig. 1:**
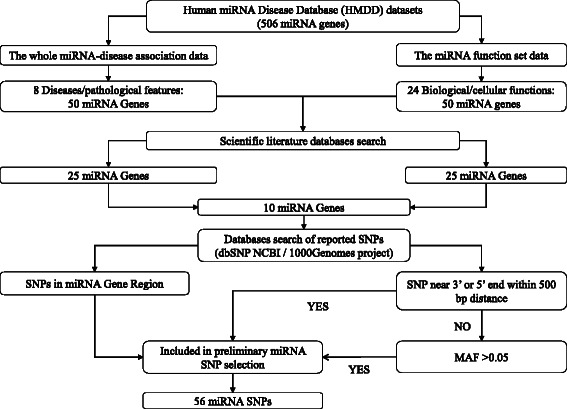
MicroRNA SNP (miR-SNP) selection algorithm using the Human miRNA Disease Database (HMDD). This flow chart shows workflow for selection of preliminary miR-SNPs included in genotyping study. Abbreviations: dbSNP, single nucleotide polymorphism database; MAF, minor allele frequency; miRNA, microRNA; NCBI National Center for Biotechnology Information; SNP, Single nucleotide polymorphisms

Figure 1 shows the selection process we followed to determine miRNA SNPs (miR-SNPs) that could be included in our study. Two datasets, “The whole miRNA-disease association data” and “The miRNA function set data” from the human miRNA disease database (HMMDD) created by Lu et al. [41] and updated in January 2012, were used to select 8 diseases and/or pathological characteristics and 24 biological and/or cellular functions related to breast cancer (See Table 1). As shown in Fig. 1, we picked the 50 miRNA genes from each dataset that were present in the majority of selected features for inclusion in the following steps. This list was narrowed down to the 25 miRNA genes on each dataset with the strongest evidence in order to maximise the potential for identification of biologically relevant molecules using two main criteria: miRNAs involved in the largest number of selected features from each group followed by a literature search to confirm the number of publications showing significant relationships to cancer biology or the possession of known functional effects of polymorphisms within the miRNA itself. Following this, we chose 10 miRNA genes from the 25 genes on both lists, again prioritising by number of functions and publications, and conducted a search to identify SNPs using both dbSNP database from The National Center for Biotechnology Information (NCBI) [42] and 1000 Genomes project browser [43]. Final selection of SNPs was done using this algorithm: All microRNA-SNPs located inside the pre-miRNA gene were automatically included in the SNP selection. However, SNPs located outside of the pre-miRNA gene were assessed using the following criteria: miR-SNPs located up to 500bp upstream or downstream from pre-miRNA were automatically included in the SNP selection. On the other hand, SNPs located more than 500bp from the 3’ or 5’ end were chosen only if they had a previously reported minor allele frequency higher than 5% in Caucasian populations. As a result 56 microRNA SNPs were identified in this preliminary selection (Data not shown) (See Fig. 1).

**Table 1:** Selected features from the Human miRNA disease database (HMDD) included in present study

Selected diseases and/or pathological features related to breast cancer			
miRNA-disease association dataset	Human miRNA genes in dataset	1. Adenocarcinoma	5. Squamous Cell Carcinoma
	503	2. Breast Neoplasms	6. Neoplasms
		3. Carcinoma	7. Germ cell and embryonal neoplasm
	Human Diseases in dataset	4. Ductal breast carcinoma	8. Squamous cell neoplasms
	396		
Selected biological and/or cellular function in relation to Cancer			
miRNA function dataset	Human miRNA genes in dataset		
	503	1. Activation of caspases cascade	13. Cell proliferation
		2. Akt pathway	14. Chemosensitivity of tumour cells
		3. Angiogenesis	15. Chemotaxis
		4. Anti-cell proliferation	16. Chromatin remodelling
		5. Apoptosis	17. DNA Repair
		6. Cell cycle related	18. Epithelial-mesenchymal transition
	Biological and cellular functions in dataset	7. Cell death	19. Human embryonic stem cell (hESC) regulation
		8. Cell differentiation	20 Immune response
		9. Cell division	21. Immune system
		10. Cell fate determination	22. Inflammation
	43	11. Cell motility	23 miRNA tumour suppressors
		12. Cell proliferation	24. Onco-miRNAs

### Primer design

Using the MassARRAY® Assay Design Suite v1.0 software (SEQUENOM Inc., San Diego, CA, USA) we were able to create a single multiplex PCR genotyping assay containing 24 miR-SNPs from our preliminary selection (See Table 2). We designed forward and reverse PCR primers and one iPLEX® (extension) primer and verified that the mass of extension primers differed by at least 30 Da among different SNPs and by 5 Da between alternative alleles of the same marker to achieve successful marker and allele identification by mass spectrometry analysis. Primers were manufactured by Integrated DNA Technologies (IDT®) Pte. Ltd. (Baulkham Hills, NSW 2153, Australia) and primer information is shown in Table 3.

**Table 2:** List of the miRNA SNPs included in multiplex primer design using the MassARRAY® Assay Design Suite v1.0 software (SEQUENOM Inc., San Diego, CA, USA)

miRNA	Locus	Location	SNPs in gene region	MAF	Location	SNPs near 3' end	MAF	Location	SNPs near 5' end	MAF	Location
MIR210	11p15.5	568,089 - 568,198				rs1062099	0.1877	567,627	rs7395206	0.5000	568,211
									rs10902173	0.4835	570,072
MIR34A	1p36.22	9,211,727 - 9,211,836	rs35301225	NA	9,211,802	rs77585961	0.0247	9,211,468			
MIR155	21q21.3	26,946,292 - 26,946,356				rs2829801	0.4707	26,944,305	rs1547354	0.0810	26,946,709
MIR221	Xp11.3	45,605,585 - 45,605,694				rs7050391	0.0133	45,605,273	rs2858061	0.4352	45,606,132
									rs2858060	0.4991	45,607,008
									rs2858059	0.4786	45,607,190
MIR222	Xp11.3	45,606,421 - 45,606,530				rs2858061	0.4352	45,606,132	rs2858060	0.4991	45,607,008
									rs2858059	0.4786	45,607,190
MIR21	17q23.1	57,918,627 - 57,918,698				rs112394324	0.0032	57,918,251	rs1292037	0.2440	57918908
						rs3851812	0.0371	57,918,370	rs13137	0.2440	57919031
MIRLET7A1	9q22.32	96,938,239 - 96,938,318				rs10761322	0.4588	96,937,478			
						rs10739971	0.2601	96,937,680			
MIRLET7A2	11q24.1	122,017,230 - 122,017,301				rs629367	0.1451	122,017,014	rs1143770	0.4867	122,017,598
									rs562052	0.3265	122,018,550
									rs693120	0.3265	122,019,011
MIR145	5q32	148,810,209 - 148,810,296				rs55945735	0.2010	148,809,158	rs353291	0.3324	148,810,746
						rs73798217	0.0545	148,809,918

**Table 3:** Primer sequences for the miRNA SNPs included in genotyping study using multiplex PCR reaction and MALDI-TOF MS

SNP	Forward primer sequence	Reverse primer sequence	iPLEX® (Extension) primer sequence
rs2829801	ACGTTGGATGTTCCCACTCAGGCATATAGC	ACGTTGGATGTGGAGTCTATGTCCACTTCC	TGGGCTAAGCAACCA
rs7395206	ACGTTGGATGTCGGACGCCCAAGTTGGAG	ACGTTGGATGTCACACGCACAGTGGGTCT	GGTGGGCGGGCGGAG
rs73798217	ACGTTGGATGACTGGAGGTTATCAGAGAGG	ACGTTGGATGATGTGTATTCCCCAGTCTCC	ACCATTCAGTTCCTAGC
rs353291	ACGTTGGATGGTAGAGATGCCACAAGAGGG	ACGTTGGATGAAACCTTAAGTCTTCGTTCG	GGTTGTTCTCTGGCTGC
rs55945735	ACGTTGGATGGGTCTCAAACTCCTGACTTC	ACGTTGGATGCTGCATCTGAGCACTTCAAG	TGATGCCTGGCGGGGCG
rs10902173	ACGTTGGATGTCACAGGCACCTTTTCTCAG	ACGTTGGATGGAAGCCTGGGTATTAGGATG	ACCTTTTCTCAGCATCTG
rs112394324	ACGTTGGATGACTGGAGAGAGAAATTACCC	ACGTTGGATGGTTGAAACCAGAGTACATGC	CAGATACGACAGAGTGTG
rs1292037	ACGTTGGATGTACAGCTAGAAAAGTCCCTG	ACGTTGGATGGGAGGGAGGATTTTATGGAG	AACCTTTTCAAAACCCACA
rs562052	ACGTTGGATGCTCCAGGCTAGTGGAATAAC	ACGTTGGATGCTACTGCACTGATCGTGTTC	CAGATTCAAATGCCATAGCA
rs2858059	ACGTTGGATGCAGTAAGTATTTCTGGGGTG	ACGTTGGATGCTCCCATGATACAATGAAGG	TGGGGTGGATAAATGAATAG
rs1062099	ACGTTGGATGGACCCGGTCCTGATTTTAAC	ACGTTGGATGTGTGTTTCTGCCGCTTCAGT	TTTAACAGTAGACTTGAGAAG
rs10739971	ACGTTGGATGCCTAATAAGACCACTTAGTGT	ACGTTGGATGATGCACTAACATACAACGAG	CCACCTACTCATTTATCCCATG
rs2858060	ACGTTGGATGACTGTATTATCCTCAGTTC	ACGTTGGATGACTTGGGTAATCTAGCAATG	GTATTATCCTCAGTTCGTAACA
rs2858061	ACGTTGGATGGCTTTCAATACTACAAGGG	ACGTTGGATGAATGATACCTTTCATAGGGG	GTAAAACAAAAACAGGTAAGAG
rs35301225	ACGTTGGATGGGCAGTATACTTGCTGATTG	ACGTTGGATGGCTGTGAGTGTTTCTTTGGC	GGATTATTGCTCACAACAACCAG
rs1547354	ACGTTGGATGTTGCAGGTTTTGGCTTGTTC	ACGTTGGATGGGAGGTTAGTAGTCCTTCTA	TTTGATTCAACTGTTAGAAATGTG
rs13137	ACGTTGGATGAGGTGAAAGAGATGAACCAC	ACGTTGGATGAAAGCATTCCCAAAATGCTC	TCCCAAAATGCTCTATTTTAGATAG
rs10761322	ACGTTGGATGGCTTTTGGTTACTAAATCAC	ACGTTGGATGCTTCATATTTAGGAGGTAGC	GGCATATTTAGGAGGTAGCTACTAC
rs693120	ACGTTGGATGGTAGATGGCACATATAGAAA	ACGTTGGATGCATCCCTTAACTGTAAGTTC	GATTGTCAAATGAAAAGAAGAATAT
rs1143770	ACGTTGGATGCTGAACAATTTAATGCCTTC	ACGTTGGATGTTCAGTTTTACCAGAGGAAC	CCCCCATGCCTTCTGATATCTGTTGA
rs77585961	ACGTTGGATGGTTTCCTTCTCTGCAAGACG	ACGTTGGATGCACTTACTATGCAGGAAGGC	CCTACATGATGTAATACACTTACAATA
rs7050391	ACGTTGGATGGTAAGGCAGTATGATTAGGC	ACGTTGGATGGCCTCAACTGTCAAAGATTG	TGTTCATAATTATTATCAGAAGGCATA
rs3851812	ACGTTGGATGTTTTCCTCCCAAGCAAAAC	ACGTTGGATGTTCTTGCCGTTCTGTAAGTG	GGTGGAAGTGTTTTATTCTTAGTGTGA
rs629367	ACGTTGGATGTATGCAGCATTTTTGTGAC	ACGTTGGATGATTCTGTTTCCTCGGGTTAG	GGGGTCAGCATTTTTGTGACAATGGACA

### Primary multiplex PCR

Genotyping was undertaken following the iPLEX™ GOLD genotyping protocol using the iPLEX® Gold Reagent Kit (SEQUENOM Inc., San Diego, CA, USA). Primer extension reactions were performed according to the instructions for the SEQUENOM linear adjustment method included in the iPLEX™ GOLD genotyping protocol (SEQUENOM Inc., San Diego, CA, USA). All reactions were performed using Applied Biosystems® MicroAmp® EnduraPlate™ Optical 96-Well Clear Reaction Plates with Barcode (Life Technologies Australia Pty Ltd., Mulgrave, VIC, Australia) and an Applied Biosystems® Veriti® 96-Well Thermal Cycler (Life Technologies Australia Pty Ltd., Mulgrave, VIC, Australia).

### MALDI-TOF MS analysis and data analysis

A total of 12-16 nl of each iPLEX® reaction product were transferred onto a SpectroCHIP® II G96 (SEQUENOM Inc., San Diego, CA, USA) using SEQUENOM® MassARRAY® Nanodispenser (SEQUENOM Inc., San Diego, CA, USA). SpectroCHIP® analysis was carried out by SEQUENOM® MassArray® Analyzer 4 and the SpectroAcquire software Version 4.0 (SEQUENOM Inc., San Diego, CA, USA). Finally data analysis for genotype determination was done using the MassARRAY® Typer software version 4.0 (SEQUENOM Inc., San Diego, CA, USA). In order to confirm the genotypes obtained, randomly selected samples (5 each for case and control cohorts) from each genotype (*n* = 240) were validated by Sanger Sequencing to ensure accuracy of genotyping results. In all cases, the Sanger Sequencing confirmed the genotyping obtained using MassARRAY.

### Statistical analysis

Statistical analysis of genotypes and alleles was conducted using Plink software version 1.07 (http://pngu.mgh.harvard.edu/purcell/plink/) [44]. The α for p-values was set at 0.05 to determine statistically significant association with breast cancer. Genotype and allele frequencies for each miRNA SNP in our case and control populations were established and we used Hardy-Weinberg equilibrium (HWE) to evaluate deviation between observed and expected frequencies for identification of unexpected population or genotyping biases [45, 46]. We performed Chi square analysis to evaluate differences in genotype and allele frequencies between cases and controls for each independent population [47]. Finally we calculated odds ratio (OR) and obtained 95% confidence interval (CI) 95% to assess disease risk.

## Results and discussion

MicroRNAs are some of the small non-coding RNA molecules responsible for gene regulation at the translational level. They require a very complex series of nuclear and cytoplasmic processes for their synthesis and to achieve their functional effects on genes involved in key cellular functions like replication and cell differentiation [7, 10, 13]. As a result they are likely to play a role in the development and progression of cancer due to the important biological mechanisms they regulate [12, 15, 16]. They have been shown to have different roles in various types of cancer including breast cancer [18, 24]. Breast cancer is a cause for concern since recent reports by the IARC show it has very high incidence and mortality rates around the world [28]. Analysis of single nucleotide polymorphisms in well-defined case control cohorts has provided information on miR-SNPs involved in the pathophysiology of different types of cancer including breast cancer [25–27, 29–33]. Therefore we selected a panel of 24 miR-SNPs related to 9 miRNA genes previously identified to play a role in breast cancer to genotype in our Australian Caucasian breast cancer case control populations. Genotyping of our selected miRNA variants in the GRC-BC cohort showed six of them to be non-polymorphic (rs73798217, rs112394324, rs35301225, rs1547354, rs7050391 and rs3851812) and another five of the chosen miR-SNPs failed to successfully deliver genotypes (rs2829801, rs7395206, rs2858059, rs1143770 and rs77585961).

On the other hand, genotype and allele frequencies of the remaining 13 miR-SNPs in the GRC-BC cases and controls showed these to be closely similar to those found in Hapmap for Caucasian populations and they were also in Hardy Weinberg Equilibrium (HWE) (*p* > 0.05). Ultimately, however, chi-square analysis of genotyping results of 12 SNPs located in relation to seven miRNAs (MIR210, MIR221, MIR222, MIR21, MIRLET7A1, MIRLET7A2 and MIR145) showed no significant differences for genotype and allele frequencies between cases and controls in our GRC-BC population (See Tables 4, 5, 6, 7, 8 and 9).

**Table 4:** Allele and genotype frequencies for miRNA SNPs in MIR210 obtained from the GRC-BC population

	rs1062099							rs10902173						
	Allele			Genotype				Allele			Genotype			
	G (%)	C (%)	*p*-value	GG (%)	CG (%)	CC (%)	*p*-value	C (%)	T (%)	p-value	CC (%)	TC (%)	TT (%)	*p*-value
Control	300 (83.3)	60 (16.7)	0.36	126 (70.0)	48 (26.7)	6 (3.3)	0.61	203 (59.0)	141 (41.0)	0.93	63 (36.6)	90 (49.5)	29 (15.9)	0.64
Cases	263 (80.7)	63 (19.3)		106 (65.0)	51 (31.3)	6 (3.7)		216 (59.3)	148 (40.7)		63 (34.6)	77 (44.8)	32 (18.6)	
Hapmap (%)	81.7	18.3		66.8	29.8	3.4		60.4	39.6		34.8	51.2	14.0

**Table 5:** Allele and genotype frequencies for miRNA SNPs in MIR221 and MIR222 from the GRC-BC Population

	rs2858061							rs2858060						
	Allele			Genotype				Allele			Genotype			
	G (%)	C (%)	*p*-value	GG (%)	CG (%)	CC (%)	*p*-value	C (%)	G (%)	*p*-value	CC (%)	GC (%)	GG (%)	*p*-value
Control	323 (87.8)	45 (12.2)	0.43	142 (77.2)	39 (21.2)	3 (1.6)	0.38	219 (69.3)	97 (30.7)	0.75	81 (51.3)	57 (36.1)	20 (12.7)	0.35
Cases	295 (85.8)	49 (14.2)		130 (75.6)	35 (20.3)	7 (4.1)		218 (68.1)	102 (31.9)		74 (46.3)	70 (43.8)	16 (10.0)	
Hapmap (%)	86.2	13.8		80.2	12.4	7.4		72.8	27.2		62.3	20.6	17.2

**Table 6:** Allele and genotype frequencies for miRNA SNPs in MIR21 obtained from the GRC-BC population

	rs1292037							rs13137						
	Allele			Genotype				Allele			Genotype			
	A (%)	G (%)	*p*-value	AA (%)	GA (%)	GG (%)	*p*-value	T (%)	A (%)	*p*-value	TT (%)	AT (%)	AA (%)	*p*-value
Control	300 (80.6)	72 (19.4)	0.86	122 (65.6%)	56 (30.1)	8 (4.3)	0.95	299 (80.8)	71 (19.2)	0.85	122 (65.9)	55 (29.7)	8 (4.3)	0.94
Cases	274 (80.1)	68 (19.9)		110 (64.3)	54 (31.6)	7 (4.1)		276 (80.2)	68 (19.8)		111 (64.5)	54 (31.4)	7 (4.1)	
Hapmap (%)	81.0	19.0		65.4	31.1	3.4		81.0	19.0		65.4	31.1	3.4

**Table 7:** Allele and genotype frequencies for miRNA SNPs in MIRLET7A1 obtained from the GRC-BC cohort

	rs10761322							rs10739971						
	Allele			Genotype				Allele			Genotype			
	T (%)	C (%)	*p*-value	TT (%)	TC (%)	CC (%)	*p*-value	G (%)	A (%)	*p*-value	GG (%)	AG (%)	AA (%)	*p*-value
Control	225 (61.8)	139 (38.2)	0.73	76 (41.8)	73 (40.1)	33 (18.1)	0.08	243 (67.5)	117 (32.5)	0.39	89 (49.4)	65 (36.1)	26 (14.4)	0.36
Cases	207 (60.5)	135 (39.5)		59 (34.5)	89 (52.0)	23 (13.5)		223 (64.5)	123 (35.5)		74 (42.8)	75 (43.4)	24 (13.9)	
Hapmap (%)	61.9	38.1		40.9	42.0	17.2		68.1	31.9		46.4	43.3	10.3

**Table 8:** Allele and genotype frequencies for miRNA SNPs in MIRLET7A2 obtained from the GRC-BC population

	rs629367							rs562052							rs693120						
	Allele			Genotype				Allele			Genotype				Allele			Genotype			
	A (%)	C (%)	*p*-value	AA (%)	CA (%)	CC (%)	*p*-value	G (%)	A (%)	*p*-value	GG (%)	AG (%)	AA (%)	*p*-value	G (%)	A (%)	*p*-value	GG (%)	AG (%)	AA (%)	*p*-value
Control	302 (82.5)	64 (17.5)	0.72	125 (68.3)	52 (28.4)	6 (3.3)	0.88	242 (65.4)	128 (34.6)	0.76	81 (43.8)	80 (43.2)	24 (13.0)	0.57	230 (67.3)	112 (32.7)	0.77	82 (48.0)	66 (38.6)	23 (13.5)	0.21
Cases	289 (83.5)	57 (16.5)		122 (70.5)	45 (26.0)	6 (3.5)		230 (66.5)	116 (33.5)		74 (42.8)	82 (47.4)	17 (9.8)		225 (66.2)	115 (33.8)		72 (42.4)	81 (47.6)	17 (10.0)	
Hapmap (%)	84.6	15.4		75.4	18.5	6.2		68.1	31.9		46.2	43.8	10.0		68.1	31.9		46.2	43.8	10.0

**Table 9:** Allele and genotype frequencies for rs55945735 located in MIR145 obtained from the GRC-BC population

	rs55945735						
	Allele			Genotype			
	A (%)	G (%)	*p*-value	AA (%)	GA (%)	GG (%)	*p*-value
Control	193 (59.9)	129 (40.1)	0.29	61 (37.9)	71 (44.1)	29 (18.0)	0.58
Cases	182 (64.1)	102 (35.9)		60 (42.3)	62 (43.7)	20 (14.1)	
Hapmap (%)	62.8	37.2		38.3	49.1	12.7	

In contrast, we were able to determine significant differences at the allelic level for rs353291 after chi-square analysis in our GRC-BC cohort (*p* = 0.041) although no significant difference in genotype frequencies (*p* = 0.09) (See Table 10). We then proceeded to genotype this SNP in the GU-CCQ BB population and we also found the genotype and allele frequencies closely matched Hapmap frequencies for Caucasian populations and cases and controls were in HWE. Statistical analysis of genotyping in our replication population showed similar findings to those obtained in the GRB-BC cohort shown in Table 10. We were able to find significant differences in allele frequencies between cases and controls in the GU-CCQ BB population (*p* = 0.006) and also statistically significant differences at the genotype level (*p* = 0.02). Finally we calculated odds ratios for alleles in the GRC-BC and GU-CCQ BB populations to be 1.37 (CI 95%: 1.01–1.84) and 1.31 (CI 95%: 1.04–1.70) respectively, suggesting the presence of the C allele at this locus increases the risk of developing breast cancer. However it should be noted that if we consider multiple testing, our finding for the analysis of allele frequencies for both populations for this variant is not significant if we determine Bonferroni correction of the α for *p*-value to be 2.08 x 10^−3^. However these results are still potentially interesting particularly considering the similarity of allelic significance in both independent case-control cohorts and point to the need for further genotyping in extended populations.

**Table 10:** Allele and genotype frequencies for SNP rs353291 located in MIR145 obtained from the GRC-BC and GU-CCQ BB cohorts

	GRC-BC population							GU-CCQ BB population						
	Allele			Genotype				Allele			Genotype			
	A (%)	G (%)	*p*-value	AA (%)	GA (%)	GG (%)	*p*-value	A (%)	G (%)	*p*-value	AA (%)	GA (%)	GG (%)	*p*-value
Control	211 (58.6)	149 (41.4)	0.041	61 (33.9)	89 (49.4)	30 (16.7)	0.09	386 (62.7)	230 (37.3)	0.023	128 (41.6)	130 (42.2)	50 (16.2)	0.07
Cases	171 (50.9)	165 (49.1)		40 (23.8)	91 (54.2)	37 (22.0)		769 (57.2)	575 (42.8)		229 (34.1)	311 (46.3)	132 (19.6)	
Hapmap (%)	62.7	37.3		40.6	44.1	15.3		62.7	37.3		40.6	44.1	15.3	

miR-SNP rs353291 is located 450 bp upstream from the MIR145 gene, inside the miRNA 143 host gene transcript, in the long arm of chromosome 5 region 32 at position 148,810,746. To the best of our knowledge there are no previous association studies in relation to this miR-SNP and breast cancer risk in Caucasians or any populations of other ethnicities. However, MIR145 has been previously reported to play a role in cancer biogenesis and progression. Downregulation of MIR145 is a common finding previously reported in colorectal [48, 49], bladder [50], lung [51, 52] and oesophageal [53] cancers possibly leading to poor prognosis. Similarly, it has also been found to have a tumour suppressor role in breast tissue particularly in the myoepithelial/basal cell compartment and it is found to be downregulated in breast cancer tissue samples [54–56]. Research using breast cancer cell lines showed it regulates genes involved in modulation of apoptosis [57, 58]. Finally downregulation of MIR145 has been associated with a more aggressive behaviour of breast malignancies based on results performed in both breast cancer cell line and tissue samples [59, 60]. However the molecular mechanisms leading to decreased expression of MIR145 still remain unknown and our finding could potentially help to provide further knowledge on these mechanisms through further functional validation on breast cancer cell culture and/or animal/models. It is also possible that rs353291 is linked to a different nearby SNP not considered in this research that may have direct functional effects on MIR145, so additional sequencing/genotyping studies may need to be performed prior to functional assessment of the link between rs353291 and breast cancer.

## Conclusions

We were able to determine that the presence of a polymorphism in miR-SNP rs353291 is associated with an increased risk of developing breast cancer based on our findings. To the best of our knowledge, this is the first report on breast cancer risk association for this variant in individuals of Caucasian background. This finding could potentially explain the previously described role that MIR145 plays in breast cancer documented in the literature, but it requires confirmation via functional studies using cell culture or animal models. It also requires further validation on larger Caucasian population as well as in cohorts of individuals with different ethnical backgrounds before it can be translated into clinical applications used in breast cancer diagnosis or treatment.

## Abbreviations

3’-UTR: 3’ untranslated region
CI: Confidence interval
DCGR8: DiGeorge syndrome critical region gene 8
DNA: Deoxyribonucleic Acid
dNTP: Deoxynucleoside triphosphate
ddNTP: Dideoxynucleoside-5’-triphosphate
dsRBD: Double-stranded RNA binding domain
HMMDD: Human miRNA disease database
HWE: Weinberg equilibrium
IARC: International Agency for Research on Cancer
mM: Milimolar
mRNA: Messenger RNA
miRNAS: MicroRNAs
miR-SNP: MicroRNA single nucleotide polymorphism
MS: Mass spectrometry
MALDI-TOF: Matrix assisted laser desorption ionization time-of-flight
NCBI: National Center for Biotechnology Information
RISC: RNA-induced silencing complex
RNA: Ribonucleic Acid
RNase: Ribonuclease
SAP: Shrimp alkaline phosphatase
SNP: Single nucleotide polymorphisms
XPO5: Exportin-5
μM: Micromolar
μl: Microlitres
°C: Celsius
